# The Glutathione Biosynthetic Pathway of *Plasmodium* Is Essential for Mosquito Transmission

**DOI:** 10.1371/journal.ppat.1000302

**Published:** 2009-02-20

**Authors:** Joel Vega-Rodríguez, Blandine Franke-Fayard, Rhoel R. Dinglasan, Chris J. Janse, Rebecca Pastrana-Mena, Andrew P. Waters, Isabelle Coppens, José F. Rodríguez-Orengo, Marcelo Jacobs-Lorena, Adelfa E. Serrano

**Affiliations:** 1 Department of Microbiology and Medical Zoology, University of Puerto Rico, School of Medicine, San Juan, Puerto Rico; 2 Department of Parasitology, Leiden University Medical Center, Leiden, The Netherlands; 3 Department of Molecular Microbiology and Immunology, Bloomberg School of Public Health, Johns Hopkins University, Baltimore, United States of America; 4 Wellcome Trust Centre of Molecular Parasitology and Division of Infection and Immunity, University of Glasgow, Glasgow, United Kingdom; 5 Department of Biochemistry, University of Puerto Rico, School of Medicine, San Juan, Puerto Rico; Washington University School of Medicine, United States of America

## Abstract

Infection of red blood cells (RBC) subjects the malaria parasite to oxidative stress. Therefore, efficient antioxidant and redox systems are required to prevent damage by reactive oxygen species. *Plasmodium spp*. have thioredoxin and glutathione (GSH) systems that are thought to play a major role as antioxidants during blood stage infection. In this report, we analyzed a critical component of the GSH biosynthesis pathway using reverse genetics. *Plasmodium berghei* parasites lacking expression of gamma-glutamylcysteine synthetase (γ-GCS), the rate limiting enzyme in *de novo* synthesis of GSH, were generated through targeted gene disruption thus demonstrating, quite unexpectedly, that γ-GCS is not essential for blood stage development. Despite a significant reduction in GSH levels, blood stage forms of *pbggcs^−^* parasites showed only a defect in growth as compared to wild type. In contrast, a dramatic effect on development of the parasites in the mosquito was observed. Infection of mosquitoes with *pbggcs^−^* parasites resulted in reduced numbers of stunted oocysts that did not produce sporozoites. These results have important implications for the design of drugs aiming at interfering with the GSH redox-system in blood stages and demonstrate that *de novo* synthesis of GSH is pivotal for development of *Plasmodium* in the mosquito.

## Author Summary

The antioxidant systems of malaria parasites (*Plasmodium spp*.) are potential targets for the development of antimalarials. The glutathione (GSH) redox system constitutes one of the *Plasmodium* primary lines of defense against damage caused by reactive oxygen species and other forms of chemical stress. GSH is synthesized *de novo* by the sequential action of gamma-glutamylcysteine synthase (γ-GCS) and GSH synthase (GS). Biochemical studies have suggested that parasite survival depends on functional *de novo* GSH synthesis. Using reverse genetics we interrupted the GSH biosynthetic pathway in the rodent malaria *Plasmodium berghei* by disrupting the *pbggcs* gene. The mutation caused minor changes in parasite growth in the mammalian host but development in the mosquito was completely arrested at the oocyst stage. These results suggest that the GSH biosynthetic pathway, while essential for mosquito stage development, is not an appropriate target for antimalarials against blood stages of the parasite.

## Introduction

*Plasmodium* infection leads to increased oxidative stress in both the vertebrate and mosquito hosts. The high proliferation rate of parasites results in the production of large quantities of toxic redox-active by-products. Reactive oxygen species (ROS) are generated within the infected RBC (iRBC) as a result of degradation of hemoglobin in the food vacuole of the parasite [1],[2]. In addition, ROS arise from the production of nitric oxide and oxygen radicals produced by the host's immune system in response to iRBC bursting and merozoite release [1],[3]. In the *Anopheles* mosquito vector, nitric oxide species and ROS are produced in response to invasion of midgut epithelial cells by the parasite [4]–[6] suggesting the requirement of efficient defense mechanisms to protect against oxidative damage. A detailed study of the *Plasmodium* genome reveals the absence of genes encoding the antioxidant enzymes catalase and glutathione peroxidase [7],[8]. The lack of a glutathione peroxidase gene raises doubts about the relevance of the glutathione (GSH) pathway in detoxification of oxidative stress in *Plasmodium*. However, supportive of a role for GSH metabolism in the detoxification process are the observations that the *P. falciparum* glutathione S-tranferase enzyme, which conjugates GSH to other molecules via the sulfhydryl group, displays peroxidase activity [9]. The *Plasmodium* GSH pathway, in conjunction with the thioredoxin redox system, could indeed act as a primary line of defense against oxidative damage [10].

To date, the role of the *Plasmodium* GSH antioxidant system has only been studied in the context of the erythrocytic stages [2],[8]. GSH is a thiol-based tripeptide implicated in a variety of cellular processes, including detoxification of xenobiotics and protection against ROS [11],[12]. Additional roles ascribed to GSH based on biochemical studies in *Plasmodium* iRBC include serving as cofactor for enzymes such as glutathione-S-transferase and as reducing agent for ferriprotoporphyrin IX, the toxic by-product of hemoglobin digestion [13]. Evidence has been presented that *P. falciparum* does not utilize GSH from the host RBC since the parasite membrane is neither permeable to host GSH nor γ-glutamylcysteine [14],[15]. *Plasmodium* is therefore thought to be dependent on its own GSH biosynthetic pathway. GSH is synthesized in *Plasmodium* by consecutive reactions facilitated by the enzymes γ-glutamylcysteine synthetase (γ-GCS) and glutathione synthetase (GS), independently of GSH biosynthesis in the host RBC, which becomes inactive after invasion [12], [14], [16]–[18]. However, Platel *et al.* [19] hypothesized that host GSH can be transported into the *P. berghei* food vacuole via hemoglobin-containing endocytic vesicles, based on data showing that GSH can detoxify the toxic ferriprotoporphyrin IX inside the parasite's food vacuole.

γ-GCS catalyzes the rate limiting step during GSH biosynthesis [20] and is inhibited in both *P. falciparum and P. berghei* by the generic γ-GCS inhibitor L-buthionine-(S,R)-sulphoximine (BSO) resulting in reduced GSH levels and, depending upon BSO concentration, in parasite death [19],[21]. These results are consistent with the expectation that *de novo* synthesis of GSH by *Plasmodium* is essential for parasite development within the iRBC. Given the oxidative environment of the iRBC it has been proposed that enzymes involved in parasite GSH biosynthesis are promising targets for the development of novel antimalarial agents [1],[2],[21].

In this study we analyzed the GSH biosynthetic pathway using reverse genetics. Following targeted gene disruption of the single copy gene encoding γ-GCS in *P. berghei* (*pbggcs*) we assessed the role and essential nature of *de novo* synthesis of GSH in parasite growth and development within the RBC and the mosquito midgut. Unexpectedly, γ-GCS is not required for blood stage development but *de novo* synthesis of GSH appears to be essential to complete sporogonic development of the parasite in the mosquito vector. These observations have important implications for the design of drugs aiming at interfering with the GSH redox-system in blood stages and may help to further unravel the functional role of GSH during development of malaria parasites in the mosquito.

## Results

### Disruption of the γ-GCS–encoding *pbggcs* gene does not prevent blood stage development

**Figure 1:**
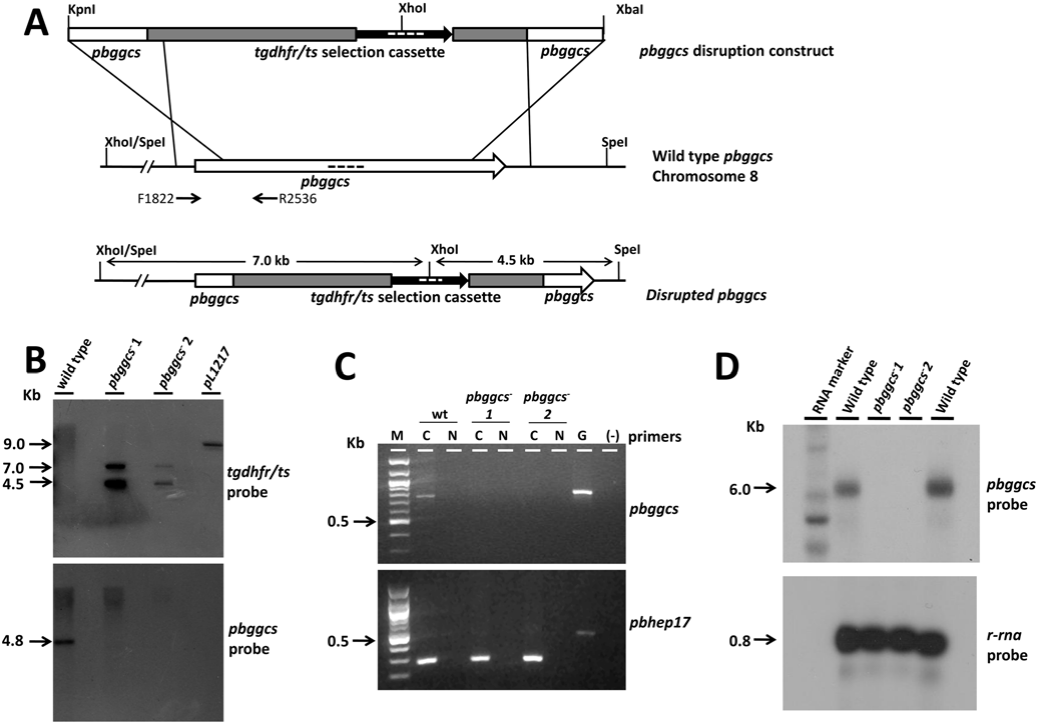
Disruption of the *pbggcs* gene. (A) Diagrammatic representation of the *pbggcs* replacement vectors (top), the *pbggcs* genomic locus (center), and the predicted integration event (bottom). The *pbggcs-ko* vectors contained the *tgdhfr/ts* selectable cassette flanked by 5′ and 3′ fragments of the *pbggcs* gene. Dashed lines inside the coding regions of *pbggcs* and *tgdhfr/ts* represent the probes used in Southern blot analysis (see B). Arrows represent primers used for PCR analysis of expression (see C). (B) Southern analysis of SpeI/XhoI digested gDNA from wild type and *pbggcs^−^* parasites. The two expected DNA fragments of 4.5 kb and 7.0 kb were obtained after hybridization with a *tgdhfr*/*ts* specific probe (left panel) demonstrating correct integration of the *pbggcs-ko* vector. Hybridization with the *pbggcs* specific probe shows the absence in the *pbggcs^−^* parasites of the 4.5 kb fragment that corresponds to wild type *pbggcs*. pL1217: *pbggcs* disruption construct. (C) Reverse transcriptase PCR analysis (RT-PCR) showing absence of *pbggcs* transcripts in *pbggcs^−^* parasites. For each cDNA synthesis reaction (C) controls included: no reverse transcriptase (N), gDNA (G), and no DNA (−). Hep17 primers were included in the RT-PCR reaction to rule out the presence of gDNA in the cDNA samples. (D) Northern analysis of mRNA of wild type and *pbggcs^−^* parasites. Hybridization with a *pbggcs*-specific probe shows the absence of *pbggcs* transcripts in *pbggcs^−^* parasites. As a loading control the Northern blot was hybridized with a ribosomal RNA probe (primer L647).

To examine the role of γ-GCS in *P. berghei* growth and development, the gene encoding γ-GCS (*pbggcs* PB001283.02.0) was disrupted using standard genetic modification technologies. In two independent experiments (exp. 866 and 985), parasites of the reference line 507cl1 (*P. berghei* ANKA strain expressing GFP; ANKA wt GFP) were transfected with the DNA-construct aimed at disruption through double cross-over integration (Fig. 1A). One parasite clone from each transfection experiment (*pbggcs^−^*1; *pbggcs^−^*2) was analyzed. Correct integration of the constructs into the parasite genome was confirmed by Southern analysis of digested DNA (Fig. 1B) and field inverted gel electrophoresis (FIGE) separated chromosomes (results not shown). The absence of *pbggcs* mRNA in blood stages of *pbggcs^−^1* and *pbggcs^−^2* was demonstrated by PCR analysis of cDNA and Northern analysis of mRNA of blood stage parasites (Fig. 1C and 1D).

### *P. berghei* blood stages can grow and develop *in vivo* in spite of low levels of intracellular GSH

**Figure 2:**
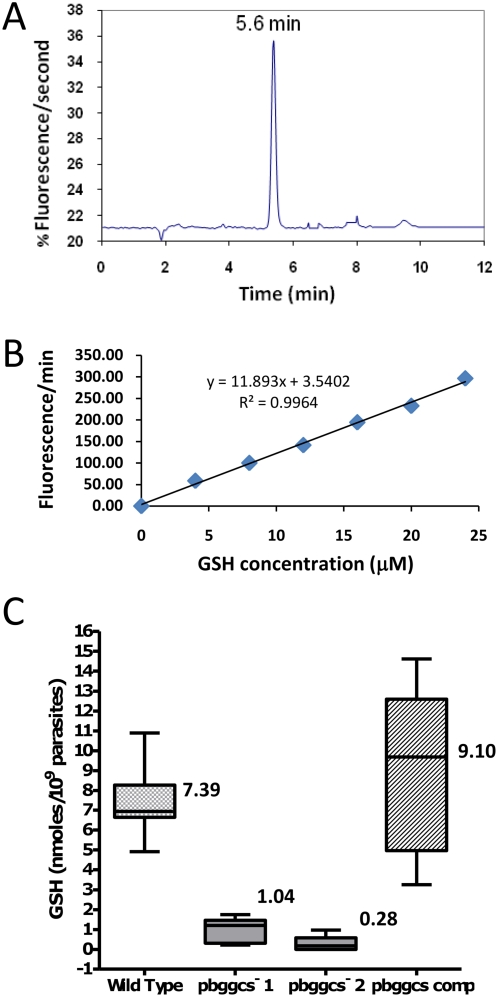
Total glutathione (GSH) levels in blood stages of wild type, *pbgccs^−^*, and *pbggcs-comp* parasites. Intracellular GSH levels were determined by HPLC with a fluorescence detector as described in Materials and Methods. (A) Typical chromatogram of *P. berghei* parasite extracts showing the signal for total GSH (retention time 5.34 min). (B) Representative calibration curve using GSH standards in a range between 4 and 24 µM. (C) Comparison of total GSH concentration of WT, *pbggcs^−^*1, *pbggcs^−^*2, and *pbggcs-comp* parasites. GSH levels were determined in extracts of purified blood stages obtained from mice with asynchronous infections. A One Way ANOVA test shows statistically significant differences in GSH concentration between *pbggcs^−^* parasites and wild type (P<0.0001) and between *pbggcs^−^* and *pbggcs-comp* parasites (P<0.0001). Numbers on boxes represent the mean concentration of GSH. Bars represent the standard error of the mean. Wild type n = 20, *pbggcs^−^*1 n = 16, *pbggcs^−^*2 n = 22, *pbggcs-comp* n = 16.

Given that γ-GCS catalyzes the rate-limiting step in GSH *de novo* biosynthesis, we predicted that disruption of the *pbggcs* locus would abolish parasite GSH levels. Parasite extracts were analyzed by HPLC for total GSH concentrations. Glutathione levels were significantly reduced (P<0.001) in blood stages of both *pbggcs^−^1* (1.0 nmol/10^9^ parasites, n = 16) and *pbggcs^−^2* (0.2 nmol/10^9^ parasites, n = 22) as compared to wild type parasites (7.4 nmol/10^9^ parasites, n = 20) (Fig. 2). Thus, the disruption of *pbggcs*, caused a significant reduction but incomplete depletion of GSH. These results demonstrate that *P. berghei* blood stages can grow and develop *in vivo* with minimal levels of GSH. The much higher concentrations found in wild type parasites are not required for survival in the RBC.

### Disruption of *pbggcs* has a minor effect on blood stage proliferation

**Figure 3:**
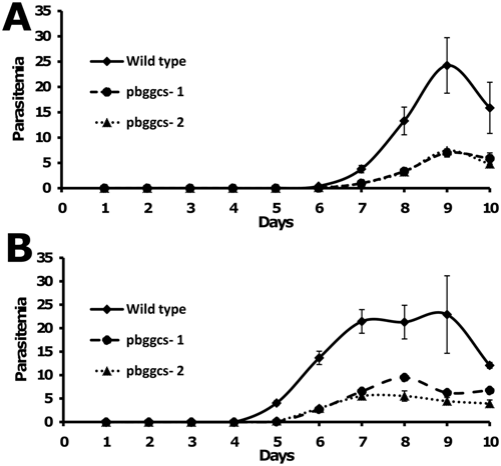
Proliferation of wild type and *pbgccs^−^* parasites in mice. The course of parasitemia was determined in groups of 5 mice infected with either 2×10^2^ (A) or 2×10^3^ (B) parasites and followed for 10 days. Each point represents the mean parasitemia. Bars represent standard errors of the mean.

When the mutant parasite lines were cloned by limiting dilution, we observed a minor decrease in the growth rate of the mutant parasites as compared to wild type. After injecting mice with a single wild type parasite, parasitemias reached 0.5–2% at 8 days post infection (d.p.i.) (mean = 8.0; st.dev. = 0; n = 6). The same level of parasitemia was reached by parasites of the mutant lines one day later (mean = 8.7; st.dev.  = 0.5; n = 6). These results suggest a delay in growth of *pbggcs^−^* parasites as compared to wild type. To further examine the potential effect of the absence of γ-GCS on growth rate, the course of parasitemia in groups of mice infected with 2×10^2^ or 2×10^3^ mutant or wild type parasites was followed in a second set of experiments (Fig. 3). The rate and extent of parasite multiplication was reduced in the mutant parasites. Therefore, although γ-GCS is not essential for growth and multiplication of blood stage *Plasmodium in vivo*, the absence of γ-GCS expression affects parasite growth.

### γ-GCS is essential for mosquito stage development of malaria parasites

**Figure 4:**
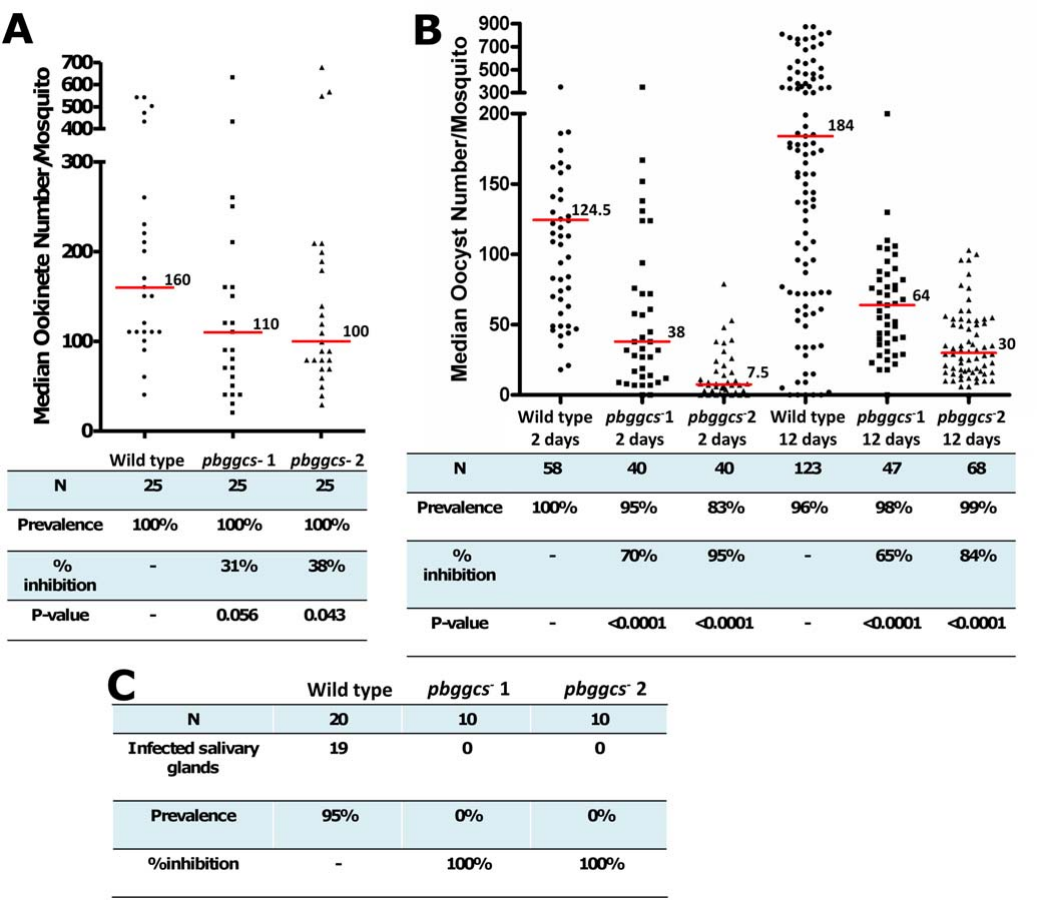
Development of *pbggcs^−^* ookinetes, oocysts, and sporozoites in *A. stephensi* mosquitoes. (A) Ookinete numbers were determined by light microscopy analysis of midguts dissected at 24 h.p.i. Horizontal lines indicate the median number of ookinetes for each group. Statistical significance was determined by Mann Whitney U Test, α = 0.05. (B) Oocyst numbers were determined on dissected midguts at 2 and 12 d.p.i. At day 2 p.i., oocysts were counted with the aid of a fluorescence microscope after incubation of midguts with an antibody against the ookinete/young oocyst surface protein Pbs21. At day 12 p.i., oocysts were stained with mercurochrome and counted using a light microscope. Horizontal lines indicate median oocyst number for each group. Statistical significance was determined by Mann Whitney U Test, α = 0.05. (C) Sporozoite infection of salivary glands was determined by dissection at day 18 p.i. and analysis of GFP-expressing sporozoites using a fluorescence microscope. Results are shown as the number of salivary glands infected with sporozoites. (N) Sample size; Prevalence: Percentage of infected mosquitoes/tissues in each group; Percent inhibition: [((control median parasite number – transfectant median parasite number)/control median parasite number)×100].

To date, *Plasmodium* GSH synthesis and metabolism have been studied only during blood stage development. To evaluate the effect of *pbggcs* disruption on the development of mosquito stages, *Anopheles stephensi* mosquitoes were infected with *pbggcs^−^* parasites and total ookinete numbers present in the mosquito midgut were determined 24 hrs post infection (h.p.i.). Ookinete numbers were reduced by 30–40% as compared to wild type parasites (n = 25) in both mutant clones (*pbggcs^−^1*: 31%, P = 0.056, n = 25; *pbggcs^−^2*: 38%, P  = 0.043, n = 25) (Fig. 4A).

**Figure 5:**
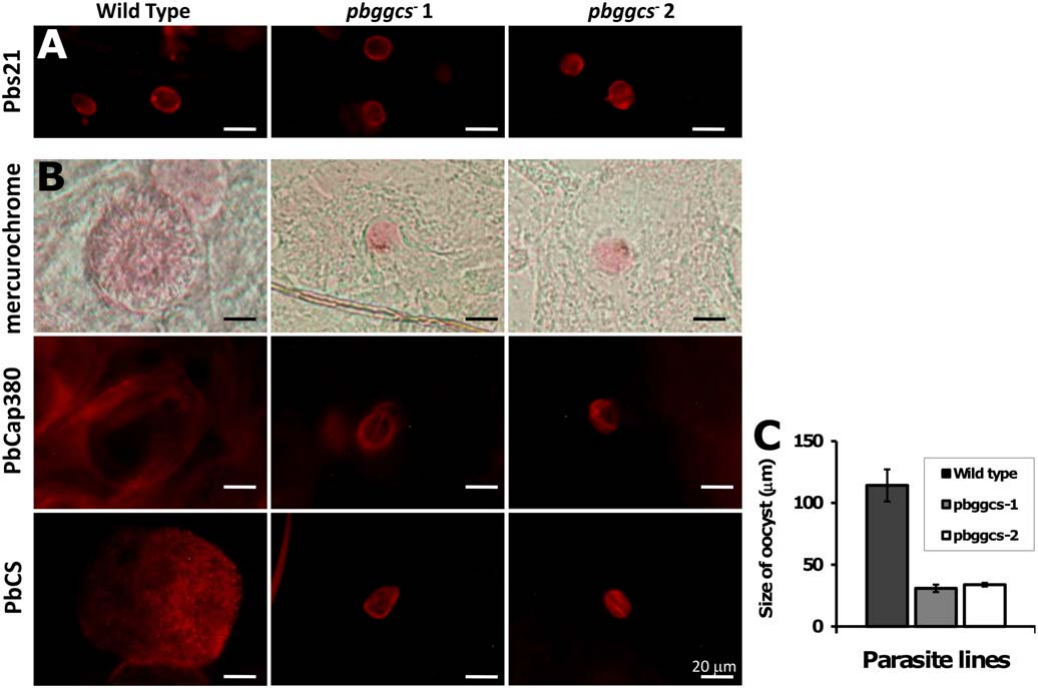
Development of *pbggcs^−^* oocysts in *A. stephensi* mosquitoes. (A) Oocysts on dissected midguts at 2 d.p.i., reacted with antibody Mab 13.1 against the ookinete/young oocysts surface protein Pbs21. Oocysts were detected after incubation with a Rhodamine Red™-X goat anti-mouse IgG (H+L) secondary antibody (Invitrogen). No differences in the reactivity pattern of oocysts from *pbgccs* ^−^ and wild type parasites were observed. (B) Oocysts on dissected midguts at day 12 p.i. stained with 0.2% mercurochrome (upper panel), antibodies against PbCap380 in the oocyst capsule (center panel) and antibodies against the surface protein of sporozoites PbCS (Mab 3D11). Antibody-stained oocysts were detected by indirect fluorescence microscopy using Rhodamine Red™-X goat anti-mouse IgG (H+L) or Texas Red®-X goat anti-rabbit IgG (H+L) secondary antibodies (Invitrogen), respectively. The *pbggcs^−^* oocysts produced the PbCap380 protein but are strongly reduced in size compared to wild type (see also C). Anti-CS staining shows that, although CS is produced, 12 day old oocysts lack the typical CS staining pattern of wild type oocysts. (C) Oocyst size on dissected midguts 12 d.p.i.

Oocyst development was examined at 2 days p.i. to determine if *pbggcs^−^* ookinetes were able to cross the mosquito midgut wall and develop beneath the basal lamina. We used antibodies against Pbs21, a protein expressed on the surface of ookinetes and young oocysts to assess early oocyst number and morphology (Fig. 4B). Pbs21-positive oocysts counts revealed that the median oocyst numbers were reduced by 70% in *pbggcs^−^1* (P<0.0001, n = 40) and by 95% in *pbggcs^−^2* (P<0.0001, n = 40) when compared to wild type parasites. Oocysts of both wild type and *pbggcs^−^* parasites showed a similar distribution of Pbs21 (Fig. 5A). Analysis of the number of mature mercurochrome stained oocysts at day 12 p.i. showed that median oocyst numbers were reduced by 65% in *pbggcs^−^1* (P<0.0001, n = 47) and by 84% in *pbggcs^−^2* (P<0.0001, n = 68) (Fig. 4B). This comparable reduction in oocyst numbers at days 2 and 12 p.i. indicates that the disruption of the *pbggcs* gene results in an early effect on oocyst development, most probably by reducing the number of ookinetes that reach the basal lamina and successfully transform into oocysts. Analysis of the mercurochrome stained *pbggcs^−^* oocysts at day 12 p.i. revealed the absence of internal structures typical of wild type parasites during sporozoite formation. In addition, these oocysts showed a significant size reduction (Fig. 5C).

The capsule protein CAP380 [22] and the circumsporozoite protein CS [23], expressed during mid to late oocyst development and sporozoite formation, respectively, were assessed in the *pbggcs^−^* oocysts by immunofluorescence analysis (IFA). Antibodies to both proteins stained *pbggcs^−^* oocysts at day 12 p.i. Reactivity with anti-PbCap380 revealed normal formation of the oocyst capsule and confirmed the reduction in the size of *pbggcs^−^* oocysts previously observed after mercurochrome staining (Fig. 5B and 5C). The reactivity pattern of *pbggcs^−^* oocysts with PbCS was clearly different from that of wild type oocysts (Fig. 5B). The normal stippling staining characteristic of the formation of sporozoites was absent in *pbggcs^−^* oocysts. The lack of internal structures in mercurochrome-stained oocysts, the absence of the characteristic sporozoite staining pattern with anti-CS antibodies, and the reduction in size indicate a defect in oocyst development and sporozoite formation.

**Figure 6:**
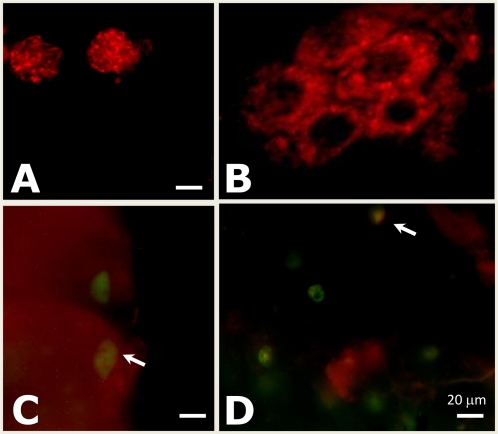
Labeling of mitochondria in live *pbgccs^−^* oocysts using MitoTracker Red. Midguts from *A. stephensi* infected with either wild type or *pbggcs^−^* parasites were dissected at day 12 p.i. and stained with MitoTracker Red (Molecular Probes). (A) Midgut containing wild type oocysts expressing GFP. Note that in this merge image of GFP and MitoTracker fluorescence, GFP fluorescence was completely quenched by the MitoTracker fluorescence. (B) Uninfected midgut (MG). Note the distinctive staining pattern of mitochondria in the gut cell surrounding the nucleus. (C, D) Midguts containing oocysts from *pbggcs^−^* 1 and *pbggcs^−^* 2 parasites, respectively. Note in this merged images of GFP and MitoTracker fluorescence, some oocysts show a partial staining with MitoTracker (arrows) while others do not stain. All panels are merged except for panel B.

It is known that mitochondria are especially sensitive to oxidative stress and that reduced GSH levels can affect the function of mitochondria as a result of oxidative damage [24],[25]. To probe mitochondria integrity, oocysts were stained at day 12 p.i. with MitoTracker, a dye which detects an intact mitochondrial membrane potential. A distinct and bright staining pattern was observed in wild type oocysts (Fig. 6A). The stippled staining pattern represents the multiple mitochondria of individual sporozoites developing within the oocyst. In contrast, only a small proportion of the *pbggcs^−^* oocysts were stained with MitoTracker (Fig. 6C and 6D) and the few MitoTracker stained oocysts, showed an aberrant staining pattern characterized by a diffused staining with reduced intensity compared to the wild type oocysts.

**Figure 7:**
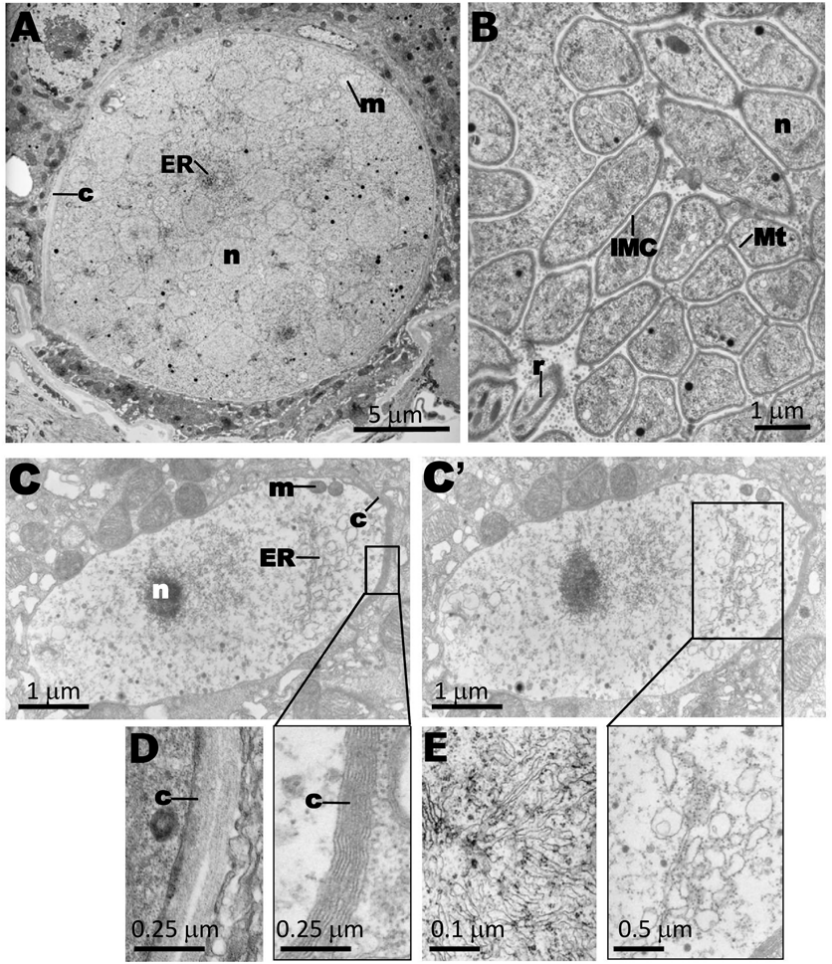
Oocysts of *pbggcs^−^* parasites: analysis of morphology by transmission electron microscopy. Electron micrographs of wild-type (A, B, D, E) and *pbggcs^−^* (C–C') oocysts at day 12 p.i. (A) Encapsulated oocyst (c, capsule) at mid-sporogony showing numerous irregular nuclear profiles, endoplasmic reticulum (ER), and mitochondria (m) localized between the nuclei (n). (B) Mature oocysts containing sporozoites. The newly formed sporozoites show the trilaminar pellicle, including the inner membrane complex (IMC), subpellicular microtubules (Mt), nucleus, and secretory organelles such as rhoptries (r). (C, C') Two serial sections of the same *pbggcs^−^* oocyst revealing the absence of internal structures characteristics of parasites undergoing sporogony (see A). The extracellular capsule is present but shows a more irregular thickness compared to wild type oocysts (see D). The nucleus is pycnotic and ER elements are largely vacuolized, compared to the branched and spiraled network of ER in the wild type oocyst (see E). (D) Morphology of capsule of wild-type oocysts. (E) ER of wild type oocysts.

To further analyze the morphology of the *pbggcs^−^* oocysts, thin-section transmission electron microscopy was performed on oocysts 12 days p.i. (Fig. 7). Compared to wild type, most of the *pbggcs^−^* oocysts exhibited a pattern of necrosis with degenerated organelles dispersed in an electron-lucent cytoplasm. Sparse mitochondria were visible in contrast to wild-type, characterized by an increased amount of mitochondria at the onset of sporozoite formation. Rough endoplasmic reticulum was abundantly present in wild type parasites, whereas the density of this organelle in *pbggcs^−^* oocysts was strongly reduced. Also the nuclei of *pbggcs^−^* oocysts were sparse and pycnotic. Despite the highly aberrant morphology, the *pbggcs^−^* oocysts showed a capsule resembling that of wild type oocysts, which is in agreement with IFA data (Fig. 5B). However, in some sections of *pbggcs^−^* oocysts, heterogeneities in the thickness of the capsule were observed (Fig. 7) whereas in wild type oocysts this structure was more homogenous. Together, these data reveal that the development of parasites lacking the expression of γ-GCS is halted during development of the oocysts at the onset of sporozoite formation.

To confirm that *pbggcs^−^* oocysts are incapable of producing viable sporozoites, salivary glands from infected mosquitoes were dissected and analyzed on days 18–25 p.i. (data from 25 days p.i. not shown). None of the salivary glands from mosquitoes infected with *pbggcs^−^* parasites (n = 20) contained sporozoites (Fig. 4C). Moreover, feeding infected mosquitoes on naïve mice at day 20 p.i. did not result in infection (data not shown). Thus, oocyst development in parasites lacking expression of γ-GCS is halted, preventing the development of viable sporozoites and interrupting transmission.

### Genetic complementation of *pbggcs^−^* parasites restores parasite development in the mosquito

**Figure 8:**
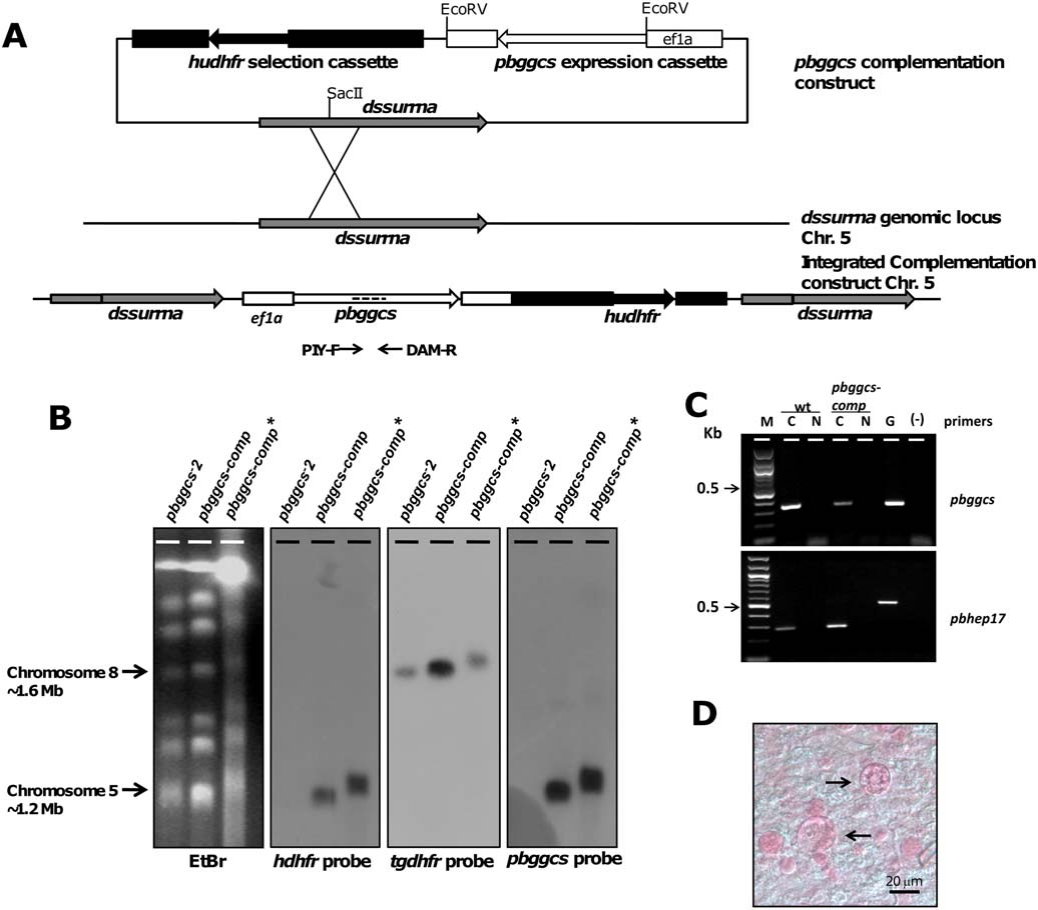
Genetic complementation of the *pbggcs^−^* parasites with the wild type *pbgccs* gene. (A) Diagrammatic representation of the construct (*pbggcs-comp*) used *for* complementation (top), the *dssurrna* genomic locus used for targeted integration of the construct (center), and the locus after integration of the construct by single cross-over recombination (bottom). The *pbggcs-comp* vector contains the *hdhfr* selectable marker and the *dssurrna* targeting sequence. Dashed lines represent the probes used for Southern analysis (see B). (B) Southern analysis of CHEF separated chromosomes from *pbggcs^−^, pbggcs-comp,* and pbggcs-comp* parasites (asterisk indicates complemented parasites after mosquito passage). Chromosomes were hybridized to different probes showing correct integration of the *pbggcs-comp* plasmid in chromosome 5, which contains the target *dssurrna* locus. (C) Reverse transcriptase PCR analysis showing the presence of *pbggcs* transcripts (345 bp fragments) in wild type and in *pbggcs-comp* parasites and the absence of *pbggcs* transcripts in *pbggcs^−^* parasites. For the PCR reaction, the specific *pbggcs* primers PIY-FM/DamR were used (see A). cDNA was synthesized from total mRNA and control reactions described in Fig. 1C. (D) Presence of maturing oocysts (stained with mercurochrome) in midguts from mosquitoes infected with *pbggcs-comp* parasites. Note the internal structure developing inside the oocysts, indicative of segmentation during sporogony and formation of sporozoites.

We used a reverse genetics approach to complement *pbggcs^−^* parasites with a wild type copy of *pbggcs*. The *pbggcs^−^* parasites (*pbggcs^−^2*) were transfected using a construct that contains the *pbggcs* gene under the control of the strong, constitutive *eef1a* promoter (Fig. 8A) [26]. In addition, this plasmid contains the *human dihydrofolate reductase* (*hdhfr*) selection cassette and the D-type small subunit rRNA (*dssurrna*) as targeting sequence for integration into the parasite genome by single cross-over recombination. Resistant parasites were selected by treatment of mice with WR99210 [27]. Correct integration of the complementation plasmid into the *dssurrna* genomic locus of these parasites (*pbggcs-comp* parasites) was confirmed by Southern analysis of contour clamped homogeneous electric field (CHEF) separated chromosomes (Fig. 8B). Detection of *pbggcs* mRNA by PCR analysis of cDNA from *pbggcs-comp* parasites confirmed successful expression of the introduced *pbggcs* gene in blood stage forms (Fig. 8C).

*A. stephensi* mosquitoes were allowed to feed on mice infected with *pbggcs-comp* parasites. Mosquito midguts were dissected 12 days p.i. and oocysts morphology was analyzed by light microscopy following mercurochrome staining. As shown in Fig. 8D, in *pbggcs-comp* parasites the morphology of oocysts was similar to that of wild type, both with respect to size and the formation of sporozoites within oocysts. The complemented parasites displayed increased GSH levels (9.1 nmol/10^9^ parasites, n = 16) when compared to *pbggcs^−^* parasites, demonstrating successful restoration of GSH biosynthesis to levels equivalent to those of wild type parasites (Fig. 2). When mosquitoes infected with *pbggcs*-*comp* parasites were allowed to feed on naïve mice, mice became infected (data not shown). Analysis of the genotype of *pbggcs-comp* parasites after mosquito transmission showed only the complemented genotype, confirming that passage to the mammalian host is only possible in the presence of *pbggcs* expression (Fig. 8B). In summary, these results show that the complementation with a functional copy of *pbggcs* restored the development of oocysts and sporozoites of the *pbggcs^−^* parasites and GSH synthesis and confirm that the arrested oocyst development phenotype and the blocking of transmission observed for the *pbggcs^−^* parasites is the result of the disruption of the *pbggcs* gene.

## Discussion

The data presented herein conclusively demonstrate that *P. berghei* parasites do not require the *de novo* synthesis of GSH for asexual development in mice. These results contrast the observed growth inhibition of *P. falciparum in vitro* following administration of the generic γ-GCS inhibitor BSO [21] and *in vivo* in *P. berghei* [19]. The highly significant reduction in parasite GSH levels resulting from disruption of the *pbggcs* gene caused only a relatively minor effect on the growth rate of intraerythocytic stages. Perhaps the low GSH levels still detected in the *pbggcs^−^* parasites are sufficient to support growth within the RBC. This GSH could be derived from the iRBC and transported to the parasite food vacuole via hemoglobin-containing endocytic vesicles, as proposed by Platel and others [19]. Evidently, GSH levels found in blood stages of wild type parasites are not essential for survival.

It could be argued that γ-GCS is not the only target of BSO in *P. berghei,* as has been proposed for *Trypanosoma brucei* [28], where supplementary GSH did not prevent death as a result of BSO treatment but was able to rescue the lethal effects of a γ-GCS gene knockdown. Although the molecular basis for strain dependent sensitivity of blood stage *P. falciparum* to BSO is unknown [29], our findings might lead one to anticipate a difference in dependence on the *de novo* GSH synthesis of blood stages of different species of *Plasmodium*. *Plasmodium vivax* and *P. berghei*, for example, preferentially infect reticulocytes [30], which contain higher levels of GSH than normocytes [31]. The γ-GCS deletion mutants should provide further insight into the role of the *de novo* synthesis of GSH and its ability to serve as a viable drug target to prevent blood stage development.

Although our results show that blood stages can survive and multiply without expression of γ-GCS, we observed a clear growth delay of the blood stages of *pbggcs^−^* parasites. This growth delay may be the result of a prolonged cell cycle, the production of fewer daughter merozoites within the schizonts, reduced invasion efficiency, or reduced proliferation in normocytes as compared to reticulocytes. Interestingly, a longer cell cycle has been reported for *Saccharomyces cerevisiae* and T98G glioblastoma cells that were depleted of GSH [32],[33]. This growth delay was detected during the G1 and S phase transition, when cells prepare for DNA replication, and it has been suggested that the slower cell cycle could provide sufficient time to repair the damage caused by the increased oxidative stress after GSH depletion. Likewise, a prolonged cell cycle as a response to decreased GSH-levels could endow *Plasmodium* blood stages with enough time to repair any critical damage as a consequence of a reduced GSH concentration. However, because GSH in the cell has additional functions apart from its role as a general thiol redox buffer, further detailed studies on cell cycle, invasion of and survival in erythrocytes of different ages are needed for more insight into the mechanism underlying the reduced growth rate of *P. berghei* blood stages as a result of low GSH levels.

In contrast to the relatively minor effect in blood stage development, disruption of *pbggcs* had a dramatic effect on parasite growth in the mosquito, completely blocking transmission. The reason for this differential requirement for endogenously-sourced GSH may lie in the potential for the profound drop in parasite GSH levels to be replaced by reducing agents from either the parasite or the iRBC. However, comparable compensatory sources might not be available in the mosquito. Furthermore, in the midgut, *Plasmodium* is initially extracellular. Levels of reactive oxygen species (ROS) are known to increase in both the midgut and hemolymph of *Anopheles gambiae* mosquitoes following a *P. berghei*-infected blood meal, as part of the innate immune response [6],[34]. Parasites lacking one of the arms of its redox defense might be less able to survive insect immunity. Alternatively, in the absence of GSH *de novo* synthesis, mosquito stages of *Plasmodium* may simply fail to deal with the intrinsic increase in oxidative stress associated with the endogenous accumulation of ROS by-products of cellular metabolism during ookinete and sporogonic development. In *Plasmodium* gametocytes and ookinetes, the number of mitochondria increases, their structure is changed, and their activity is intensified [35]–[37]. Mitochondria are one of the main ROS producers in the cell, therefore one of the first organelles to be adversely affected by a poorly restrained increase in these harmful radicals. The damage that ROS can induce in this organelle, which include mitochondrial DNA mutations as well as lipid and protein peroxidation, is limited by GSH [25]. The significant reduction of GSH in *pbggcs^−^* mutants could result in accelerated mitochondrial damage and loss of function, further production of ROS and ultimately, death by apoptosis [38]. Therefore, the augmented activity of mitochondria during the development of *P. berghei* ookinetes and oocyst might lead to an increased dependence on GSH for maintaining ROS reduction and redox homeostasis.

Interestingly, it has been shown using reverse genetics that disrupting genes encoding other proteins associated with the parasite redox systems, such as thioredoxin peroxidase from *P. falciparum* (*pftpx-1*) and *P. berghei* (*pbtpx-1*), are also not essential for blood stage development [39],[40]. In addition, depletion of the antioxidant plasmoredoxin, an enzyme from the thioredoxin superfamily, which links the thioredoxin and the glutathione redox system [41], showed no detectable effect on parasite development in the RBC [42]. The redundancy in function of multiple members of the thioredoxin family has been postulated as a possible explanation for these results [42]. Furthermore, functional redundancy stemming from the presence of both thioredoxin and glutathione redox systems as a defense against oxidative stress might also explain the fact that the individual systems are not essential for asexual development [41]–[43]. This may be comparable to the multiple redundant pathways for hemoglobin digestion [44] and erythrocyte invasion [45] developed by the parasite to optimize growth and survival within the host RBC.

The observation that fully developed infectious *pbggcs^−^* sporozoites were not produced and that infected mosquitoes were unable to transmit the infection to naïve mice are extremely relevant to the field. Disruption of the *pbggcs* gene resulted in attenuated development of oocysts and subsequent failure to produce sporozoites, as evidenced by the production of an oocyst capsule that is highly heterogeneous as to thickness, the loss of mitochondria membrane potential in the mutant oocysts, and abnormal patterns of expression of the circumsporozoite (CS) protein [23]. A significant reduction in the amount of mature *pbggcs^−^* ookinetes in the mosquito midguts compared to wild type was also detected followed by an even greater reduction in numbers of oocysts at the basal lamina. The combined results from mosquito stages of *pbggcs^−^* parasites point to a continuing failure of the *pbggcs^−^* mutant to develop in the vector. This spectrum of defects is quite consistent with a mutant that has a reduced capacity to deal with ROS due to an absence of *de novo* synthesis of GSH. In agreement with the present findings, both protracted and altered cell cycles have been reported for *S. cerevisiae*, *Arabidopsis thaliana* and T98G glioblastoma cells when available GSH levels were critically depleted either through drug treatment or genetic manipulation [32],[33],[46]. Further studies are needed to determine whether a comparable cell cycle defect underlies the deleterious effects of the absence of γ-GCS in *Plasmodium* mosquito stages. The fact that blood stage parasites could survive without γ-GCS raises some doubts on its potential use as a target in the development of new drugs against parasite blood stages in man. A clarification of such issues requires resolution of the precise roles of GSH, its biosynthetic pathway and the interplay with other redox systems. The role of the parasite GSH-redox system in mosquito stages has not been previously addressed and our results encourage further studies aimed at unraveling the critical role of this pathway for the survival of the parasite in its vector.

## Materials and Methods

### DNA constructs and parasite transfection

A gene targeting construct was made to knock out the *pbggcs* gene (PB001283.02.0). To replace the gene, 5′ and 3′ flanking regions of the *pbggcs* gene were cloned up- and downstream of the selection cassette of plasmid pL0001 (Malaria Research and Reference Reagent Resource Center: MR-4) that contains the *Toxoplasma gondii dihydrofolate reductase–thymidylate synthase* (*tgdhfr*/*ts*) selectable marker cassette. An 885 bp DNA fragment from the 5′ region of the *pbggcs* locus was PCR amplified with primers 2751 (5′-GGGGTACCCGTACATGTACGCATATATTATACA-3′; KpnI site is underlined) /2752 (5′-CCCAAGCTTGGCAATCATTTCCACTTTCTAAATTCATC-3′; HindIII site is underlined) and cloned into KpnI/HindIII digested pL0001 vector to obtain pL0001-*5′pbggcs*. Additionally, the complete *pbggcs* ORF including the 3′UTR was amplified with primers 2562 (5′-CATGCCATGGATGGGTTTTCTAA AAATTGGAACTCC-3′; KpnI site is underlined) /2563 (5′- CGGGGTACCTGGTGTGTATATACCAAACCGTTTC-3′; KpnI site is underlined), cloned into the TOPO TA vector (Invitrogene) and then sequenced to obtain *pbggcs*-TOPO plasmid. To generate the 3′ targeting region, a fragment of 754 bp was digested from *pbggcs*-TOPO plasmid with the HincII/ NotI enzymes and cloned into EcoRV/NotI pL0001-*5′pbggcs* digested plasmid to create the *pbggcs* disruption vector, pL1217. For transfection, pL1217 was linearized with KpnI/SacII restriction enzymes and transfected into *P. berghei* purified schizonts of line 507cl1 [47]. Briefly, this line, ANKA wt-GFP, expresses GFP under the control of the constitutive *eukaryotic elongation factor 1A* (*eef1aa*) promoter and has been selected by Fluorescence Activated Cell Sorter (FACS) sorting based on GFP expression as described by Janse *et al*. [47]. Transfection, selection and cloning of *pbggcs^−^* parasites was carried out as previously described [27]. Correct integration of pL1217 into the *pbggcs* genomic locus was confirmed by standard Southern blot analysis of digested genomic DNA using *tgdhfr/ts* and *pbggcs* specific probes (Fig. 1B). The 921 bp *tgdhfr/ts* probe was obtained by digesting pL0001 with SalI and the 833 bp *pbggcs*-specific probe was obtained by digesting the *pbggcs*-TOPO plasmid with SpeI/NheI. Hybridizations were performed according standard methods. Expression of the *pbggcs* gene was analyzed by PCR amplification of reverse transcribed mRNA. Total RNA of blood stages obtained from mice with asynchronous infections was isolated using RNA STAT-60 (Tel-Test Inc.) according to manufacturer's specifications. Complementary DNA (cDNA) was synthesized with the Superscript II RNase H- Reverse Transcriptase (Invitrogen) following manufacturer's recommendations. PCR's were carried out on 1 µl of synthesized cDNAs using the *pbggcs* specific primers F1822 (5 TTAACGGTTTTCTGTAAATGC3)/R2536 (5′- TTCTTCTTATTTTCATACAATGCTC-3′) which amplifies 746 bp of the 5′ region of the *pbggcs* gene including the 5′UTR. A control PCR was included to exclude potential contamination with gDNA by using primers directed to the two exons of the *P. berghei hepatocyte erythrocyte protein 17* gene (*pbhep17*) homologue of the *pyhep17* [48].

Plasmid pL0009 (MR4) was used as a backbone for the *pbggcs-*complementation construct. This plasmid contains the *hdhfr* selectable marker which confers resistance to pyrimethamine and the antimalarial WR99210 and targeting sequence for integration into the *c*- or *d*-*ssurrna* by single cross-over recombination. First, the *green fluorescence protein* (*gfp*) *mutant 3* gene (BamHI/XbaI) of pL0017 was exchanged for the *e-gfp* gene of plasmid pEGFP-NI (Clonetch, subcloned SacII/NotI in pBluescript-SK) to create the ef-eGFP vector. Then, the coding sequence of the *pbggcs* gene including the 3′ UTR of *pbggcs*-Topo plasmid was digested with KpnI/NcoI and cloned into the ef-eGFP plasmid to obtain pL1136. Finally, the EcoRV fragment of pL1136 plasmid (which contains the *pbggcs* expression cassette) was cloned into the pL0009 vector to obtain the *pbggcs* complementation plasmid (*pbggcs*-*comp*). Both expression cassettes (*pbggcs* and *hdhfr*) are in the same orientation in this vector. Plasmid *pbggcs*-*comp* was linearized with SacII and transfected into *pbggcs2^−^* parasites as previously described [27]. Transfected parasites were selected after a four day treatment with 16 mg/kg body weight of the drug WR99210. Correct integration of the complementation construct into the c/d-ssurrna on chromosome 5/6 was verified by Southern analysis of chromosome separated by Contour-clamped Homogeneous Electric Field (CHEF) electrophoresis using standard procedures. Southern analysis was performed using the *pbggcs* and *tgdhfr/ts* specific probes described above. Expression of the *pbggcs* gene in *pbggcs-comp* parasites was analyzed by reverse transcription PCR (RT-PCR) and Northern blot analysis of total mRNA of blood stage parasites. RT-PCR analysis was done using the *pbggcs* specific primers PIY-F (5′- TATAAAGATGTAAATACAG-3′)/DAM-R (5′- CATTCCAAAAAACATTGCATC-3′). Northern blots were hybridized to the *pbggcs*-specific probe used in the Southern analysis, as described above. As a loading control the Northern blot was hybridized with a ribosomal RNA probe (primer L647) [49].

Constructs pL0001, pL0009 and pL0017 and parasite line 507cl1 can be obtained from the Malaria Research and Reference Reagent Resource Center (MR4; http://www.malaria.mr4.org/).

### Mice

Random-bred Swiss albino CD-1 female mice (Charles River Laboratories, Wilmington, MA, USA), 6–8 weeks old, weighting 20 to 35 g at the time of primary infection were used throughout the study. They were kept in a room with a temperature of 22°C and a 12h light /12h dark cycle. All studies involving laboratory animals were performed in accordance with the regulations of the US Institutional Animal Care and Use Committee (IACUC) and the regulations of the Dutch Experiments on Animals Act.

### Determination of GSH levels in blood stage parasites

Parasite intracellular GSH levels were determined using a modified HPLC method previously described [modified from 50 and 51].

#### Parasite isolation

*P. berghei* infected blood (0.8–1.0 mL) was harvested by heart puncture from mice with parasitemias between 5% and 15%. White blood cells and platelets were removed by using a Whatman® CF11 cellulose column [52]. Infected RBCs were centrifuged and washed with saline solution and subsequently lysed with saponin (0.15%) for 10 minutes at 4°C. Free parasites were centrifuged, washed with saline solution and resuspended at a final concentration of 50×10^6^/100 µL in a buffer containing 3.5 mM MgCl_2_, 110 mM KCl, 40 mM NaCl, 20 mM Hepes, 6 mM EDTA pH 7.4 and a protease inhibitors cocktail [53]. Prior to total GSH extraction, parasite lysis was achieved by three successive freeze (liquid nitrogen) and thaw (37 °C) cycles.

#### GSH extraction

Parasite extracts were treated with 12.5 mM dithioerythritol (DTE) for 5 minutes to reduce total GSH, which include GSH, GSSG and GS-protein in the sample. Phosphoric acid (1.0 M) was then added for protein precipitation. After centrifugation, 150 µL of the sample were transferred to a 1.5 mL tube containing 100 mg of sodium bicarbonate. To this solution, one hundred microliters (100 µL) of 0.1 M Tris-HCl pH 8.00 and 2 µL monobromobimane (MBrB) were added. The derivatization was carried out at room temperature for 5 minutes in the dark and was terminated by adding 20 µL of 60% phosphoric acid. Samples were centrifuged at 14, 000 RPMs for two minutes and the supernatant was filtered (Millipore 0.22 µm filter) at 5,000 RPMs for two minutes.

#### Total GSH measurement

Samples were loaded on to a Hewlett Packard HP ODS Hypersyl column (5.00 µm, 200×4.0 µm) and analyzed as MBrB derivatives with a Hewlett Packard 1050 Series HPLC. The compounds were eluted at a flow rate of 1.2 mL/min with mobile phase (0.25% acetic acid, 0.25% phosphoric acid, 9% acetonitrile, and 90.5% water). After 7 minutes, a gradient was established to 5% mobile phase and 95% acetonitrile up to 8.5 min and the flow rate was increased to 1.7 mL/min for 0.5 minutes. At 9 minutes, the system was returned back to 100% mobile phase and 1.2 mL/min. Total GSH in the effluent was measured using a Hewlett Packard 1046A Programmable fluorescence detector (excitation 388 nm; emission 491 nm). Fluorescence intensity was measured and the area of the GSH peak (not peak height) was used to quantify the amount of total GSH when compared to the calibration curve. GSH calibration curves were ran every time a set of samples were analyzed using six different concentrations between 4 and 24 µM GSH.

### Determination of growth of asexual blood stages *in vivo*

The asexual multiplication rate *in vivo* was analyzed by determination of the parasitemia at day 7–9 after injecting mice (i.v.) with a single parasite (during the procedure of cloning of the mutants by limiting dilution). The percentage of erythrocytes infected with a single parasite of reference lines of the ANKA strain of *P. berghei* consistently ranges between 0.5–2% at day 8 after infection, resulting in a mean multiplication rate of 10 per 24h. In addition, groups of mice (5) were infected with 20 to 200 or 200 to 2000 parasites of either ANKA wt-GFP or *pbggcs^−^* parasites (*pbggcs^−^*1 or *pbggcs^−^*2). Parasitemia (P = % of infected erythrocytes) was determined by microscopic examination of Diff Quick-stained thin smears of tail blood every day during a period of 10 days.

### Analysis of the mosquito stages of *pbggcs^−^* parasites

*Anopheles stephensi* female mosquitoes were allowed to feed on mice infected with *P. berghei* ANKA wt-GFP and *pbggcs^−^* parasites (clones 1 or 2). Feeding was performed when the exflagellation rate of male gametocytes in the infected blood was between 1 and 2 per 10 fields [54]. In each experiment, mosquitoes were fed on one mouse and mosquitoes were kept at 21°C, feeding [55].

#### Ookinetes

Midguts (n = 25) filled with the blood bolus were dissected 24 hours p.i. and homogenized in 50 µl 1× PBS. From the homogenate, 5 µl were smeared and fixed on a slide by incubation in methanol for 10 minutes at −20°C. Parasites were counted on each slide after Giemsa staining and total numbers of ookinetes per midguts were calculated.

#### Oocysts

To determine the numbers of oocysts per mosquito, midguts (n≥40) were dissected 2 days and 12 days p.i., fixed in 4% paraformaldehyde, and blocked with 3% bovine albumin serum. The blood bolus was removed from midguts dissected at day 2 p.i. and the “empty” midguts incubated with an antibody specific for the surface protein, Pbs21 (Mab 13.1) [56], of ookinetes and young oocysts. The antibody-stained oocysts at day 2 p.i. were detected and counted by fluorescent microscopy (400× magnification) after incubation with a Rhodamine Red™-X goat anti-mouse IgG (H+L) (Invitrogen). Oocysts from midguts dissected at day 12 p.i. were stained with 0.2% mercurochrome for 10 minutes at room temperature and counted using a light microscope (400× magnification). In addition, midguts dissected at day 12 p.i. were stained with antibodies against two proteins, PbCap380 [22] and PbCS Mab 3D11 [23]. Antibody-stained oocysts were analyzed by fluorescence microscopy (400× magnification) with Rhodamine Red™-X goat anti-mouse IgG (H+L) or Texas Red®-X goat anti-rabbit IgG (H+L) (Invitrogen), respectively. The mean size of oocyst was determined at day 12 p.i. by measuring the diameter of 30 to 80 GFP-expressing oocysts from 5 to 8 individual mosquitoes using a fluorescence stereomicroscope Fluo Combi III (Leica, NL) and the Leica Application Suite AF software (Leica, NL).

To analyze the membrane potential of mitochondria of the oocyst stage, midguts dissected at day 12 p.i. were incubated with the 100 nM MitoTracker® Red (Molecular Probes) for 10 minutes at room temperature. To stain mosquito midgut epithelial cells and establish the difference in staining pattern between oocysts and midgut epithelial cells, midguts were stained with 100 nM MitoTracker for a period of 30 minutes at room temperature. Stained oocysts or midguts were analyzed using a fluorescence microscope at a magnification of 400× and 1000× oil immersion, excitation at 579 nm and emission at 600 nm.

#### Sporozoites

To analyze sporozoite production, mosquito salivary glands were dissected between 18 and 25 days p.i. and homogenized in 50 µl of 1× PBS. Salivary gland sporozoites were detected by light and fluorescence (GFP) microscopy. To analyze infectivity of sporozoites, infected mosquitoes (n = 50) were fed on naïve mice (2 per experiment) for a period of 15–20 minutes. The presence of parasites in the mice blood stream was monitored daily at day 6 to day 12 after feeding of the mosquitoes by analysis of Giemsa stained thin smears from tail blood.

### Transmission electron microscopy analysis of oocysts

After infection of *A. stephensi* mosquitoes with ANKA wt-GFP and *pbggcs^−^* parasites, midguts were dissected at day 12, fixed in 2.5% glutaraldehyde (Electron Microscopy Sciences; EMS, Hatfield, PA) in 0.1 M sodium cacodylate buffer (pH 7.4) for 1 h at room temperature, and processed as described by [57] using a Philips 410 Electron Microscope (Eindhoven, the Netherlands) under 80 kV.

### Statistical analysis

One way Anova (GraphPad prism 4.03, GraphPad Software, Inc. La Jolla, CA) or Mann Whitney U (STATVIEW 5.0 software, SAS Institute, Cary, NC) tests were used to determine statistical differences between parasite samples. In both cases, a P value of ≤ 0.05 was used to establish statistical significance.

### Accession numbers

The *P. berghei ggcs* (*pbggcs*) DNA sequence was retrieved from PlasmoDB (http://plasmodb.org/plasmo): PlasmoDB gene identifier PB001283.02.0. Complete *pbggcs* DNA sequence was obtained from contig PB_RP2837.

## References

[1] Becker K, Tilley L, Vennerstrom JL, Roberts D, Rogerson S (2004). Oxidative stress in malaria parasite-infected erythrocytes: host-parasite interactions.. Int J Parasitol.

[2] Müller S (2004). Redox and antioxidant systems of the malaria parasite Plasmodium falciparum.. Mol Microbiol.

[3] Tilley L, Loria P, Foley M, Totowa RPJ (2001). Chloroquine and other quinoline antimalarials.

[4] Dong Y, Aguilar R, Xi Z, Warr E, Mongin E (2006). *Anopheles gambiae* immune responses to human and rodent *Plasmodium* parasite species.. PLoS Pathog.

[5] Peterson TM, Luckhart S (2006). A mosquito 2-Cys peroxiredoxin protects against nitrosative and oxidative stresses associated with malaria parasite infection.. Free Radic Biol Med.

[6] Molina-Cruz A, DeJong RJ, Charles B, Gupta L, Kumar S (2008). Reactive oxygen species modulate *Anopheles gambiae* immunity against bacteria and *Plasmodium*.. J Biol Chem.

[7] Sztajer H, Gamain B, Aumann KD, Slomianny C, Becker K (2001). The putative glutathione peroxidase gene of *Plasmodium falciparum* codes for a thioredoxin peroxidase.. J Biol Chem.

[8] Becker K, Rahlfs S, Nickel C, Schirmer RH (2003). Glutathione-functions and metabolism in the malarial parasite *Plasmodium falciparum*.. Biol Chem.

[9] Harwaldt P, Rahlfs S, Becker K (2002). Glutathione S-transferase of the malarial parasite Plasmodium falciparum: characterization of a potential drug target.. Biol Chem.

[10] Krauth-Siegel RL, Bauer H, Schirmer RH (2005). Dithiol proteins as guardians of the intracellular redox milieu in parasites: old and new drug targets in trypanosomes and malaria-causing plasmodia.. Angew Chem Int Ed Engl.

[11] Sies H (1999). Glutathione and its role in cellular functions.. Free Radic Biol Med.

[12] Müller S, Gilberger TW, Krnajski Z, Lüersen K, Meierjohann S (2001). Thioredoxin and glutathione system of malaria parasite *Plasmodium falciparum*.. Protoplasma.

[13] Liebau E, Bergmann B, Campbell AM, Teesdale-Spittle P, Brophy PM (2002). The glutathione S-transferase from *Plasmodium falciparum*.. Mol Biochem Parasitol.

[14] Atamna H, Ginsburg H (1997). The malaria parasite supplies glutathione to its host cell: investigation of glutathione transport and metabolism in human erythrocytes infected with *Plasmodium falciparurn.*. Eur J Biochem.

[15] Ayi K, Cappadoro M, Branca M, Turrini F, Arese P (1998). *Plasmodium falciparum* glutathione metabolism and growth are independent of glutathione system of host erythrocyte.. FEBS Lett.

[16] Lüersen K, Walter RD, Müller S (1999). The putative gamma-glutamylcysteine synthetase from *Plasmodium falciparum* contains large insertions and a variable tandem repeat.. Mol Biochem Parasitol.

[17] Birago C, Pace T, Picci L, Pizzi E, Scotti R (1999). The putative gene for the first enzyme of glutathione biosynthesis in *Plasmodium berghei* and *Plasmodium falciparum*.. Mol Biochem Parasitol.

[18] Meierjohann S, Walter RD, Müller S (2002). Glutathione synthetase from *Plasmodium falciparum*.. Biochem J.

[19] Platel DF, Mangou F, Tribouley-Duret J (1999). High-level chloroquine resistance of *Plasmodium berghei* is associated with multiple drug resistance and loss of reversal by calcium antagonists.. Int J Parasitol.

[20] Meister A (1983). Selective modification of glutathione metabolism.. Science.

[21] Lüersen K, Walter RD, Müller S (2000). *Plasmodium falciparum-infected* red blood cells depend on a functional de novo synthesis of glutathione attributable to an enhanced loss of glutathione.. Biochem J.

[22] Srinivasan P, Fujioka H, Jacobs-Lorena M (2008). PbCap380, a novel oocyst capsule protein, is essential for malaria parasite survival in the mosquito.. Cell Microbiol.

[23] Enea V, Arnot D, Schmidt EC, Cochrane A, Gwadz R (1984). Circumsporozoite gene of *Plasmodium cynomolgi* (Gombak):cDNA cloning and expression of the repetitive circumsporozoite epitope.. Proc Natl Acad Sci U S A.

[24] Turrens JF (2003). Mitochondrial formation of reactive oxygen species.. J Physiol.

[25] Lash LH (2006). Mitochondrial glutathione transport: physiological, pathological and toxicological implications.. Chem Biol Interact.

[26] Franke-Fayard B, Trueman H, Ramesar J, Mendoza J, van der Keur M (2004). A *Plasmodium berghei* reference line that constitutively expresses GFP at a high level throughout the complete life cycle.. Mol Biochem Parasitol.

[27] Janse CJ, Ramesar J, Waters AP (2006). High-efficiency transfection and drug selection of genetically transformed blood stages of the rodent malaria parasite *Plasmodium berghei*.. Nat Protoc.

[28] Huynh TT, Huynh VT, Harmon MA, Phillips MA (2003). Gene knockdown of gamma-glutamylcysteine synthetase by RNAi in the parasitic protozoa *Trypanosoma brucei* demonstrates that it is an essential enzyme.. J Biol Chem.

[29] Meierjohann S, Walter RD, Müller S (2002). Regulation of intracellular glutathione levels in erythrocytes infected with chloroquine-sensitive and chloroquine-resistant *Plasmodium falciparum*.. Biochem J.

[30] Mons B (1990). Preferential invasion of malarial merozoites into young red blood cells.. Blood Cells.

[31] Sailaja YR, Baskar R, Saralakumari D (2003). The antioxidant status during maturation of reticulocytes to erythrocytes in type 2 diabetics.. Free Radic Biol Med.

[32] Sharma KG, Sharma V, Bourbouloux A, Delrot S, Bachhawat AK (2000). Glutathione depletion leads to delayed growth stasis in Saccharomyces cerevisiae: evidence of a partially overlapping role for thioredoxin.. Curr Genet.

[33] Russo T, Zambrano N, Esposito F, Ammendola R, Cimino F (1995). A p53-independent pathway for activation of WAF1/CIP1 expression following oxidative stress.. J Biol Chem.

[34] Kumar S, Christophides GK, Cantera R, Charles B, Han YS (2003). The role of reactive oxygen species on *Plasmodium* melanotic encapsulation in *Anopheles gambiae*.. Proc Natl Acad Sci U S A.

[35] Sinden RE, Killick-Kendrick R, Peters W (1978). Cell Biology.. Rodent Malaria.

[36] Krungkrai J, Prapunwattana P, Krungkrai SR (2000). Ultrastructure and function of mitochondria in gametocytic stage of *Plasmodium falciparum*.. Parasite.

[37] Hall N, Karras M, Raine JD, Carlton JM, Kooij TW (2005). A comprehensive survey of the *Plasmodium* life cycle by genomic, transcriptomic, and proteomic analyses.. Science.

[38] Tsutsui H, Ide T, Kinugawa S (2006). Mitochondrial oxidative stress, DNA damage, and heart failure.. Antioxid Redox Signal.

[39] Komaki-Yasuda K, Kawazu S, Kano S (2003). Disruption of the *Plasmodium falciparum* 2-Cys peroxiredoxin gene renders parasites hypersensitive to reactive oxygen and nitrogen species.. FEBS Lett.

[40] Yano K, Komaki-Yasuda K, Tsuboi T, Torii M, Kano S (2006). 2-Cys Peroxiredoxin TPx-1 is involved in gametocyte development in *Plasmodium berghei*.. Mol Biochem Parasitol.

[41] Becker K, Kanzok SM, Iozef R, Fischer M, Schirmer RH (2003). Plasmoredoxin, a novel redox-active protein unique for malarial parasites.. Eur J Biochem.

[42] Buchholz K, Rahlfs S, Schirmer RH, Becker K, Matuschewski K (2008). Depletion of *Plasmodium berghei* plasmoredoxin reveals a non-essential role for life cycle progression of the malaria parasite.. PLoS ONE.

[43] Kanzok SM, Schirmer RH, Turbachova I, Iozef R, Becker K (2000). The thioredoxin system of the malaria parasite *Plasmodium falciparum*. Glutathione reduction revisited.. J Biol Chem.

[44] Liu J, Istvan ES, Gluzman IY, Gross J, Goldberg DE (2006). *Plasmodium falciparum* ensures its amino acid supply with multiple acquisition pathways and redundant proteolytic enzyme systems.. Proc Natl Acad Sci U S A.

[45] Cowman AF, Crabb BS (2006). Invasion of red blood cells by malaria parasites.. Cell.

[46] Vernoux T, Wilson RC, Seeley KA, Reichheld JP, Muroy S (2000). The ROOT MERISTEMLESS1/CADMIUM SENSITIVE2 gene defines a glutathione-dependent pathway involved in initiation and maintenance of cell division during postembryonic root development.. Plant Cell.

[47] Janse CJ, Franke-Fayard B, Waters AP (2006). Selection by flow-sorting of genetically transformed, GFP-expressing blood stages of the rodent malaria parasite, *Plasmodium berghei*.. Nat Protoc.

[48] Doolan DL, Hedstrom RC, Rogers WO, Charoenvit Y, Rogers M (1996). Identification and characterization of the protective hepatocyte erythrocyte protein 17 kDa gene of *Plasmodium yoelii*, homolog of *Plasmodium falciparum* exported protein 1.. J Biol Chem.

[49] van Spaendonk RM, Ramesar J, van Wigcheren A, Eling W, Beetsma AL, van Gemert GJ (2001). Functional equivalence of structurally distinct ribosomes in the malaria parasite, Plasmodium berghei.. J Biol Chem.

[50] Mansoor MA, Svardal AM, Ueland PM (1992). Determination of the in vivo redox status of cysteine, cysteinylglycine, homocysteine, and glutathione in human plasma.. Anal Biochem.

[51] Rodriguez JF, Cordero J, Chantry C, González S, Rivera C (1998). Plasma glutathione concentrations in children infected with human immunodeficiency virus.. Pediatr Infect Dis J.

[52] Baggaley VC, Atkinson EM (1972). Use of CF 12 columns for preparations of DNA from rodent malarias.. Trans R Soc Trop Med Hyg.

[53] Birago C, Marchei E, Pennino R, Valvo L (2001). Assay of gamma-glutamylcysteine synthetase activity in *Plasmodium berghei* by liquid chromatography with electrochemical detection.. J Pharm Biomed Anal.

[54] Arai M, Billker O, Morris HR, Panico M, Delcroix M (2001). Both mosquito-derived xanthurenic acid and a host blood-derived factor regulate gametogenesis of *Plasmodium* in the midgut of the mosquito.. Mol Biochem Parasitol.

[55] Dinglasan RR, Kalume DE, Kanzok SM, Ghosh AK, Muratova O (2007). Disruption of *Plasmodium falciparum* development by antibodies against a conserved mosquito midgut antigen.. Proc Natl Acad Sci U S A.

[56] Tirawanchai N, Sinden RE (1990). Three non-repeated transmission blocking epitopes recognized in the 21 kD surface antigen of zygotes-ookinetes of *Plasmodium berghei*.. Parasite Immunol.

[57] Quittnat F, Nishikawa Y, Stedman TT, Voelker DR, Choi JY (2004). On the biogenesis of lipid bodies in ancient eukaryotes: synthesis of triacylglycerols by a *Toxoplasma* DGAT1-related enzyme.. Mol Biochem Parasitol.

