# Correction: The Glutathione Biosynthetic Pathway of *Plasmodium* Is Essential for Mosquito Transmission

**DOI:** 10.1371/annotation/69b47852-9d8d-42c5-8c6e-9f42ebda0d2f

**Published:** 2009-06-05

**Authors:** Joel Vega-Rodríguez, Blandine Franke-Fayard, Rhoel R. Dinglasan, Chris J. Janse, Rebecca Pastrana-Mena, Andrew P. Waters, Isabelle Coppens, José F. Rodríguez-Orengo, Prakash Srinivasan, Marcelo Jacobs-Lorena, Adelfa E. Serrano

Dr. Prakash Srinivasan was not included in the author byline. He should be listed as the ninth author and affiliated with Department of Molecular Microbiology and Immunology, Bloomberg School of Public Health, Johns Hopkins University, Baltimore, Maryland, United States of America. The contributions of this author are as follows: Developed pbCAP380 antibody.

